# A prospective comparison of Scheimpflug-based anterior segment imaging systems in normal and abnormal corneas: Oculus Pentacam vs. Remidio Visionix VX650

**DOI:** 10.3389/fopht.2025.1516423

**Published:** 2025-02-19

**Authors:** Sonam Kumar, Kalpa Negiloni, Parul Chawla Gupta, Ranjitha K, Neha N, Divya Parthasarathy Rao, Jyothi Damani, Ritika Thakur, Shonraj Ballae Ganesh Rao, Mona Duggal

**Affiliations:** ^1^ Advance Eye Centre, Postgraduate Institute of Medical Education and Research (PGIMER), Chandigarh, India; ^2^ Remidio Innovative Solutions Pvt Ltd, Bengaluru, Karnataka, India; ^3^ Remidio Innovative Solutions, Inc, Glen Allen, VA, United States

**Keywords:** anterior imaging, Scheimpflug imaging, cornea, Pentacam, anterior segment analyzer

## Abstract

**Purpose:**

Advancements in anterior segment imaging have significantly improved the precision of corneal diagnostics, aiding ocular conditions management. The study evaluated the agreement between two Scheimpflug devices—Pentacam and Remidio Visionix 650 (VX650)—in assessing corneal and anterior chamber parameters in patients with and without corneal abnormalities.

**Materials and methods:**

This prospective study at a tertiary eye care hospital measured pachymetry values (central corneal thickness, thinnest point), front corneal surface parameters (keratometry, curvature, astigmatism), back corneal surface parameters (elevation, curvature) and anterior chamber parameters (depth, volume, angle). Bland-Altman analysis assessed agreement between the devices, and the Intraclass Correlation Coefficient (ICC) evaluated VX650’s repeatability.

**Results:**

Of the 202 patients (mean age was 25 ± 4.33 years), 141 eyes were classified as normal and 61 as abnormal (41 keratoconus, 17 post-LASIK, 3 post-ICL). Bland-Altman analysis showed good agreement between Pentacam and VX650 for anterior segment parameters. The VX650 measured marginally thicker corneal thickness (mean difference of central corneal thickness (CCT) of -4.0 ± 6.3 µm). Statistically significant differences in apical corneal thickness were noted for post-refractive surgery patients. K1 and K2 values showed significant variations in keratoconus but were not clinically significant. Anterior chamber depth was similar between devices, though volume differences were observed post-surgery. ICC indicated good to excellent repeatability (above 0.75) for most parameters.

**Conclusion:**

Remidio Visionix VX650 showed good agreement with Pentacam for key anterior segment measurements, confirming its reliability for corneal diagnostics. Additionally, its multifunctional capabilities offer potential for streamlining workflows, especially in clinics seeking efficient, comprehensive eye assessments.

## Introduction

Recent advancements in anterior segment imaging technology have significantly enhanced eye care, enabling precise diagnosis and management of various ocular conditions. These technologies are pivotal in identifying and treating corneal abnormalities, glaucoma (especially angle-closure glaucoma), refractive errors, and optimizing contact lens fittings (1–3). Eye care professionals can obtain detailed cornea maps through corneal tomography, facilitating a deeper understanding of anterior eye conditions and improving diagnostic and treatment plans (4–8). Among these technologies, Placido disk and Scheimpflug imaging devices are indispensable for assessing corneal topography and tomography. However, despite its many advantages, the Scheimpflug-based system has some limitations, including its limited resolution to image the posterior segment and its limited utility in cataract evaluation.

Several corneal conditions such as Keratoconus and other ectatic corneal disorders present unique challenges in diagnosis and management due to their progressive nature and significant impact on visual function. Early detection is critical, as ectasia often progresses rapidly in younger populations, especially in pediatric patients where it is frequently diagnosed at moderate to advanced stages (9). Systematic corneal tomography plays a vital role in identifying subtle corneal changes and monitoring progression. Moreover, delayed interventions, such as corneal cross-linking (CXL), increase the risk of disease progression in the second eye and impose greater economic and psychological burdens (10). Improved imaging technologies are essential to address these challenges, offering high-resolution imaging and precise measurements of both anterior and posterior in a multimodal device facilitating early diagnosis and intervention, ultimately improving patient outcomes (9).

The Remidio Visionix 650 ((Luneau Technology, France, and Remidio Innovative Solutions, Bengaluru, India; VX650) is an advanced multifunctional screening and diagnostic device designed to evaluate both the anterior and posterior segment of the eye in a single, integrated system. It consists of an array of testing and imaging capabilities, including wavefront aberrometry, Scheimpflug imaging, corneal topography, retro-illumination, anterior chamber analysis, pachymetry, non-contact tonometry and fundus imaging. These functionalities enable comprehensive eye examinations to be conducted quickly and efficiently. The device is particularly useful for evaluating candidates for refractive surgery, diagnosing keratoconus, and managing other corneal conditions. It streamlines the diagnostic process by reducing the need for multiple devices and tests. By providing accurate, fast, and non-invasive measurements, the VX650 improves workflow efficiency and supports clinicians in making informed decisions in eye care.

The VX650 is a comprehensive diagnostic device that can be used in cornea clinics for corneal topography, pachymetry, and anterior segment analysis, making it an efficient tool for pre- and post-operative assessments in refractive surgery and corneal disease management. In this study, we aimed to evaluate the agreement of anterior segment variables between Pentacam (Occulus Pentacam^®^ AXL, Oculus Optikgeräte GmbH, Germany) and VX650 in patients with and without clinically diagnosed corneal abnormalities. This comparison will enhance the understanding of the VX650’s performance and help integrate its use into corneal assessment, referral, and treatment planning in clinical practice.

## Materials and methods

### Study design and ethical considerations

This prospective cross-sectional study was conducted at the Ophthalmology department of a tertiary eye care hospital, India (South Asia) over a five-month period, from October to December 2023. The study adhered strictly to the ethical guidelines of the Declaration of Helsinki and approved by the ethics committee (Ethics Approval No. IEC-INT/2023/SPI-1256).

### Study participants

Consecutive patients aged 18 and older who were referred for cornea diagnostics and scheduled for Pentacam testing as part of their routine eye care were recruited. Informed consent was obtained from all participants prior to any assessments. Participants underwent routine eye examination at the hospital, and the final clinical diagnosis based on comprehensive evaluation was recorded. Participants included those referred for Pentacam test, such as patients planning to undergo refractive surgery (constituting the normal cornea group in our study), patients who had previously undergone any type of refractive surgery, and patients with a diagnosis or suspicion of keratoconus or any corneal conditions (constituting abnormal cornea group). Exclusion criteria included patients with conditions that contraindicated or limited Pentacam test, such as active eye infections, significant cataracts, leucomatous corneal opacities, severe ocular surface disease, unstable fixation, or recent ocular surgery (any type performed within two weeks). Additionally, individuals who had worn contact lenses within two weeks preceding the study and with incomplete test result records (VX650 and/or Pentacam) were also excluded.

### Instruments used in this study

Pentacam employs a rotating Scheimpflug camera to capture a full 360-degree rotating image of the corneal surface, utilizing a monochromatic slit-light source (475 nm). (oculus pentacam). This system revolves around the optical axes of the eye, allowing for the calculation of three-dimensional (3D) values for the anterior segment. This system captures 50 images in 2 seconds, generating approximately 25,000 elevation points, which provide detailed data on corneal curvature, thickness, anterior chamber depth, and elevation maps. The VX650 is an advanced multimodal anterior-to-posterior segment diagnostic device. It merges the Hartmann-Shack wavefront aberrometer, Scheimpflug-based imaging system, Placido disk corneal topographer, non-contact tonometry, and 45-degree retinal imaging. It employs a static horizontal scan using a Scheimpflug camera, providing a pachymeter measuring range from 150 to 1300 μm with a resolution of ±10 μm. The device measures the Iridocorneal (IC) angle within a range of 0° to 60°, with a resolution of 0.1°. Pupil illumination is achieved using blue light at 455 nm, enhancing measurement accuracy. For corneal topography, the VX650 utilizes 24 placido rings to capture data from 6144 measuring points, analyzing over 100,000 data points across a diameter ranging from 0.75 mm to more than 10 mm. These features make the VX650 a comprehensive tool for accurate ocular diagnostics and management.

### Investigations

**Figure 1:**
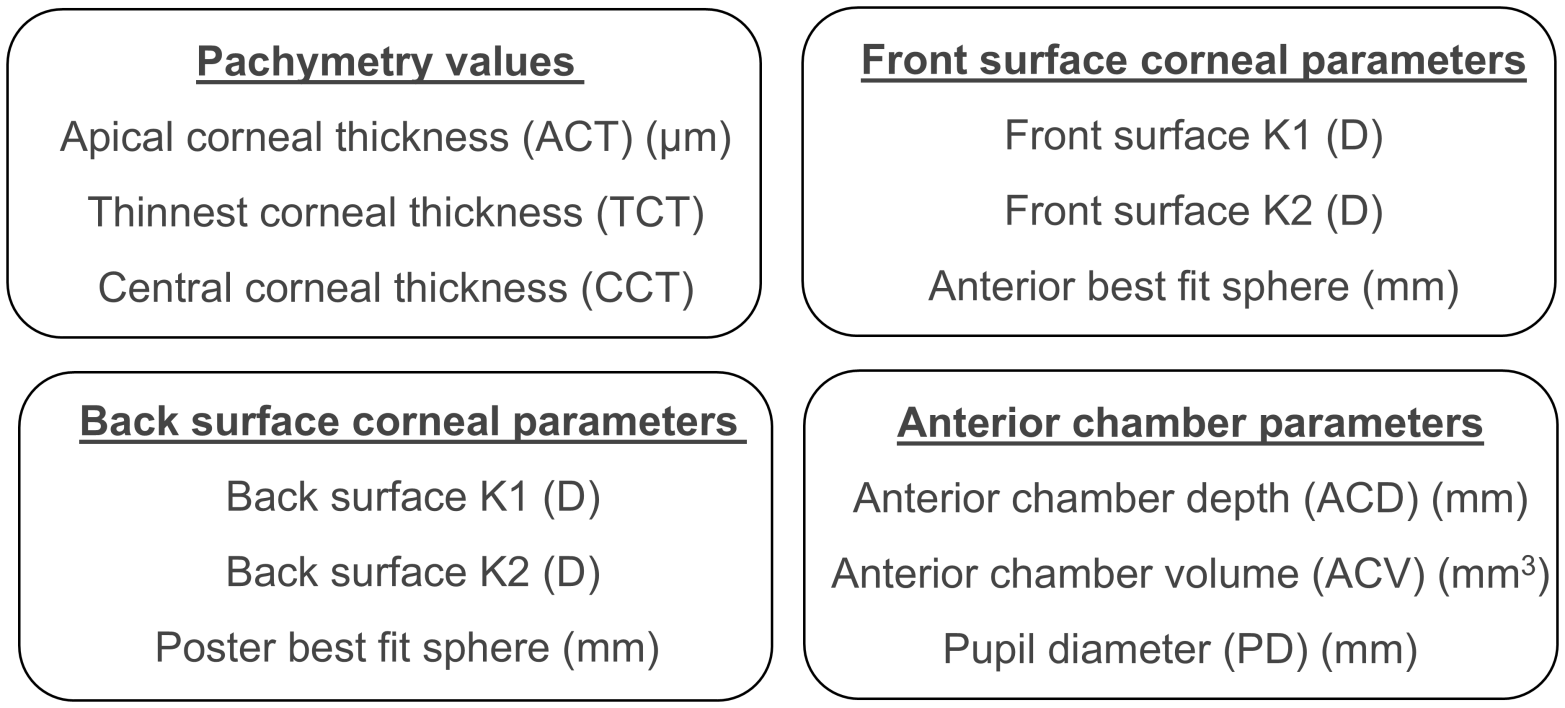
Pachymetry, anterior chamber, front corneal surface, and back corneal surface parameters compared between Pentacam and VX650.

As shown in **Figure 1**, anterior segment biometry and corneal parameters were assessed using two distinct devices based on the Scheimpflug principle: the Pentacam and the VX650. These devices provide detailed data on corneal curvature, thickness, anterior chamber depth, and elevation maps by processing extensive elevation points captured during high-resolution imaging.

### Data collection procedure

Two trained examiners performed the tests using both devices. The order of testing using both the devices was randomized. For evaluating the repeatability, a single examiner took three measurements on the same participant using the VX650 device, with a 10-minute break between each test.

### Scan quality criteria

For Pentacam scan, only those scans rated as “okay” or “high” in overall quality standard (QS) parameter were included in the study. The VX650 device provides an alert to the operator regarding the quality of the mires before capturing readings, indicating whether they are good or poor.

### Testing conditions

Both Pentacam and VX650 tests were conducted in a dimly lit room, with a 10-minute gap between sessions to minimize potential daily variations in the readings. The order of test was randomized. Participants were instructed to maintain a steady gaze on the camera’s target while ensuring their head and chin were securely positioned on the headrest and chinrest. To prevent bias, the examiners were blinded to the results of the other device. Different examiners were assigned to each device, with no access to the readings or results obtained from the other device.

### Sample size

The sample size for the study was calculated using the following formula


$$1.96*\frac{s}{\sqrt{\text{n}}}=d$$

Where, d = desired confidence interval of limits of agreement s = standard deviation of mean; difference and n = sample size. For this study, the desired confidence interval for the limits of agreement was set at 0.07, and the standard deviation (s = 0.51) was taken from a previous study by Zhang et al. (19), which compared the previous version of Visionix with Pentacam in healthy corneal conditions. The required sample size estimate was 203 participants.

Statistical analysis was conducted using R statistical software. Bland-Altman (BA) analysis was used to assess the agreement between Pentacam and VX650 values. In BA analysis, the p-value is used to determine whether the mean difference between two measurement methods is statistically different from zero. A p-value of less than 0.05 was considered statistically significant. The intraclass correlation coefficient (ICC) was calculated to assess the repeatability of measurements taken three times using the VX650. ICC values below 0.5 were considered poor, between 0.5 and 0.75 as moderate, between 0.75 and 0.9 as good and any value above 0.9 as excellent repeatability (11). Since both RE and LE correlated (r >0.85) for all parameters, data from RE was only considered for analysis.

## Results

A total of 212 patients were initially enrolled in the study. However, 10 patients were excluded due to missing variables in Pentacam and/or VX650 reports. This left 202 patients whose right-eye data were included in the analysis. Among these, 141 eyes (69.8%) were classified as the “normal group,” as they had no clinically diagnosed corneal abnormalities. The remaining 61 eyes (30.2%) formed the “abnormal group,” which consisted of 41 eyes (20.3%) diagnosed with keratoconus and 20 eyes (9.9%) from patients who had undergone refractive surgery—17 post Laser-Assisted *In Situ* Keratomileusis (LASIK) (8.4%) and 3 with Implantable Collamer Lens (ICL) (1.5%). The mean age of the study participants was 25 ± 4.33 years, ranging from 18 to 40 years.

### Agreement for pachymetry values

Corneal thickness measurements at various locations (apex, thinnest point, and central area) revealed that the VX650 device consistently provided marginally thicker readings compared to Pentacam. The mean ± standard deviation of Apical corneal thickness (ACT) measured by Pentacam across both normal and abnormal groups was 511.3 µm ± 40.96 µm, while the VX650 measured 517.29 µm ± 43.38 µm. The thinnest corneal thickness (TCT) measured by Pentacam was 507.06 µm ± 42.22 µm, compared to 510.22 µm ± 47.4 µm with the VX650. For central corneal thickness (CCT), Pentacam measured 511.58 µm ± 39.91 µm, while the VX650 measured 515.58 µm ± 43.83 µm, indicating that the VX650 produced slightly thicker measurements overall.

**Figure 2:**
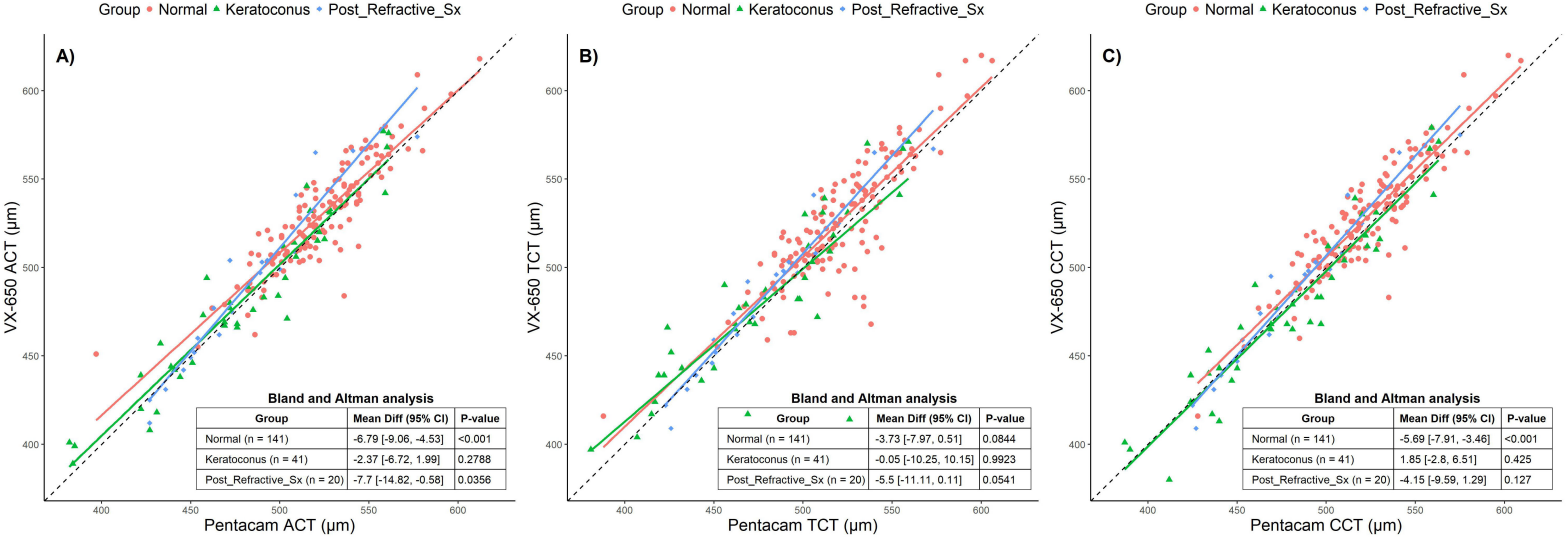
Scatter plots comparing Pentacam and VX650 pachymetry parameters across all three groups: Normal, Keratoconus, and Post-Refractive Surgery. Panel **(A)** shows apical corneal thickness (ACT), panel **(B)** shows total corneal thickness (TCT), and panel **(C)** shows central corneal thickness (CCT). The table within each plot presents the Bland-Altman mean difference (95% CI) and the p-value for each pachymetry parameter, comparing measurements from Pentacam and VX650 across the three groups.

**Figure 3:**
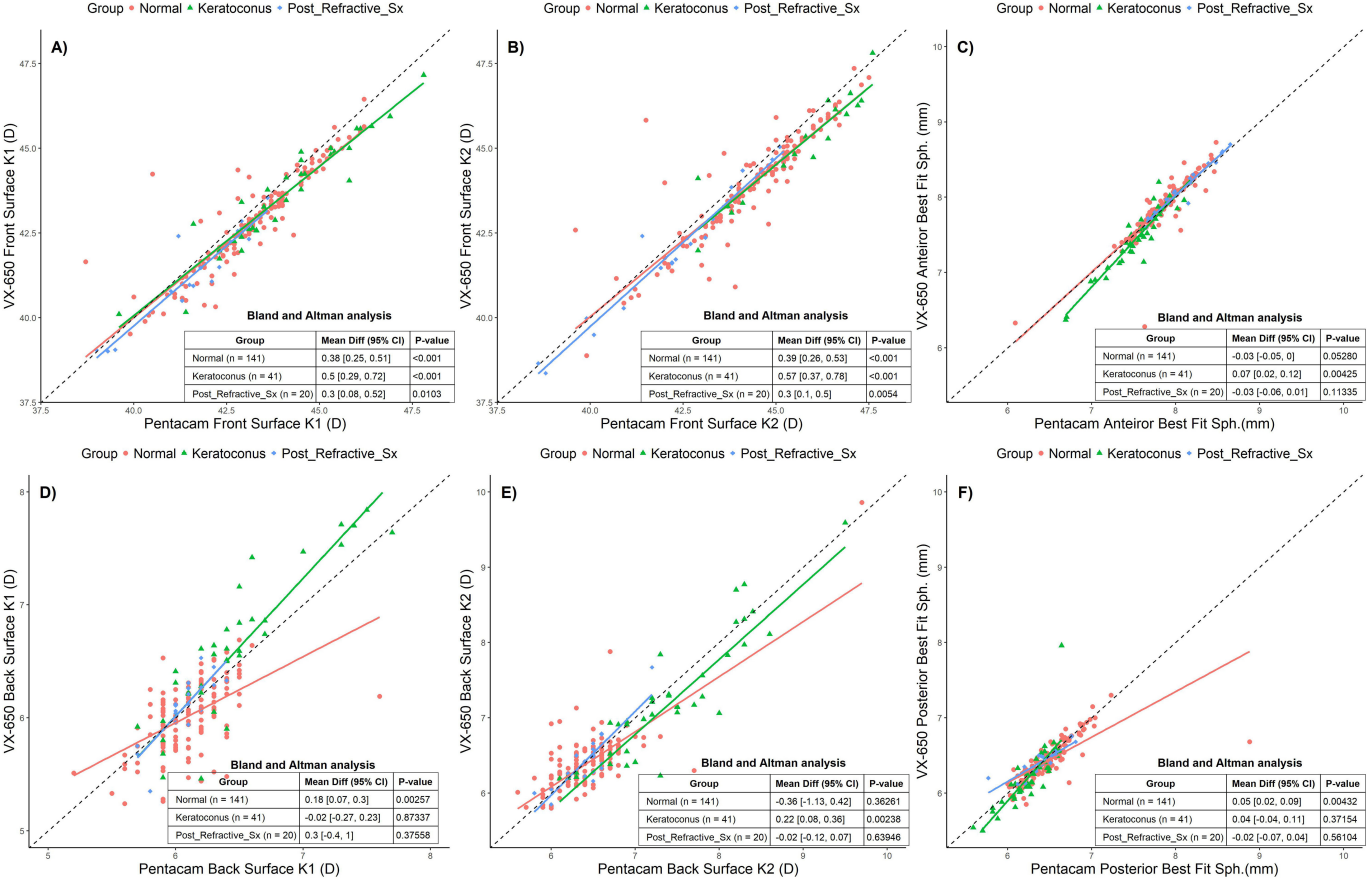
Scatter plots comparing Pentacam and VX650 for front and back surface corneal parameters across all three groups: Normal, Keratoconus, and Post-Refractive Surgery. Panel **(A)** shows anterior surface K1, panel **(B)** shows anterior surface K2, panel **(C)** shows anterior best fit sphere, **(D)** shows posterior surface K1, panel **(E)** shows posterior surface K2, and panel **(F)** shows posterior best fit sphere. The table within each plot presents the Bland-Altman mean difference (95% CI) and the p-value for each pachymetry parameter, comparing measurements from Pentacam and VX650 across the three groups.

**Figure 4:**
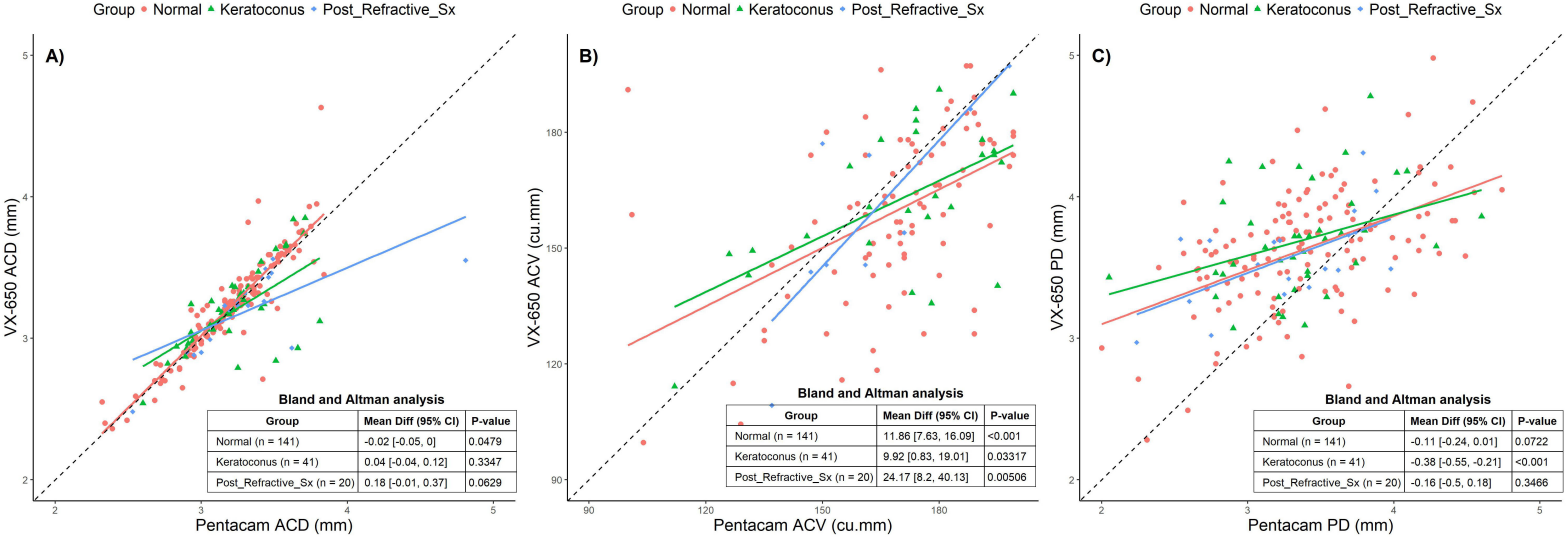
Scatter plots comparing Pentacam and VX650 for anterior chamber parameters across all three groups: Normal, Keratoconus, and Post-Refractive Surgery. Panel **(A)** shows anterior chamber depth (ACD), panel **(B)** shows anterior chamber volume (ACV), and panel **(C)** shows pupillary diameter (PD). The table within each plot presents the Bland-Altman mean difference (95% CI) and the p-value for each pachymetry parameter, comparing measurements from Pentacam and VX650 across the three groups.

**Figure 2** presents scatter plots and Bland-Altman (BA) analyses for the three groups: normal, keratoconus, and post-refractive surgery patients. The black dotted line in **Figure 2** represents the 1:1 line, data points falling on this line indicates identical measurements were made from both devices. A separate regression line is fitted for each group, and the deviation of these regression lines from the 1:1 line illustrates the differences in measurements between the two devices for each group. The Bland-Altman (BA) analysis is shown as a table insert in each subplot. Similar analyses are presented in **Figures 3**, **4**. The BA analysis in **Figure 2** revealed statistically significant deviations in ACT between the groups, especially in the post-refractive surgery group. TCT showed no statistically significant differences, while CCT demonstrated statistically significant differences in the normal group but not in others. Although statistically significant, these differences are not considered clinically significant.

### Agreement for corneal indices

The anterior surface K1 and K2 values measured using Pentacam were 43.42D ± 2.47D and 45.12D ± 3.31D, respectively, while the VX650 measured 43.02D ± 2.32D and 44.69D ± 3.32D. The posterior surface K1 and K2 values measured by Pentacam were 6.43D ± 0.43D and 6.63D ± 0.62D, while the VX650 measured 6.04D ± 0.93D and 6.83D ± 3.91D. The anterior best-fit sphere measured using Pentacam was 7.79 mm ± 0.33 mm, compared to 7.8 mm ± 0.39 mm with the VX650. The posterior best-fit sphere measured by Pentacam was 6.42 mm ± 0.32 mm, and for the VX650, it was 6.38 mm ± 0.31 mm.

The BA analyses reveal (**Figure 3**) that Pentacam and VX650 devices exhibit statistically significant differences in several parameters, particularly for Front Surface K1 and K2 values across all groups. These differences are most pronounced in Keratoconus patients, indicating that this group experiences the largest variation between devices. For the Back Surface parameters, the Normal group shows significant differences in K1, and the Keratoconus group shows differences in K2. However, many of the measurements for the Post-Refractive Surgery group did not show significant differences. While there are statistically significant differences between the two devices for certain parameters, these differences vary based on the group and the specific parameter being measured. Despite the statistical significance, the clinical impact of these variations was not significant.

### Agreement for anterior chamber parameters

The mean ACD measured by Pentacam across all groups was 3.22 mm ± 0.32 mm, while VX650 measured 3.22 mm ± 0.34 mm. The mean ACV was 188.25 mm³ ± 32.61 mm³ with Pentacam and 175.56 mm³ ± 31.91 mm³ with VX650. The PD measured by Pentacam was 3.52 mm ± 0.82 mm, compared to 3.69 mm ± 0.53 mm with VX650.

**Figure 4** presents scatter plots and BA analyses for all three groups: Normal, Keratoconus, and Post-Refractive Surgery. The BA analyses indicate that ACD exhibited a small but statistically significant difference in the Normal group only. ACV showed statistically significant differences across all groups, with the largest deviation seen in post-refractive surgery patients. PD exhibited significant differences in the Keratoconus group, where VX650 measurements were smaller than those from Pentacam. While there are measurable differences between the devices, particularly for ACV and PD, these differences may not be clinically significant.

### Repeatability analysis

**Figure 5:**
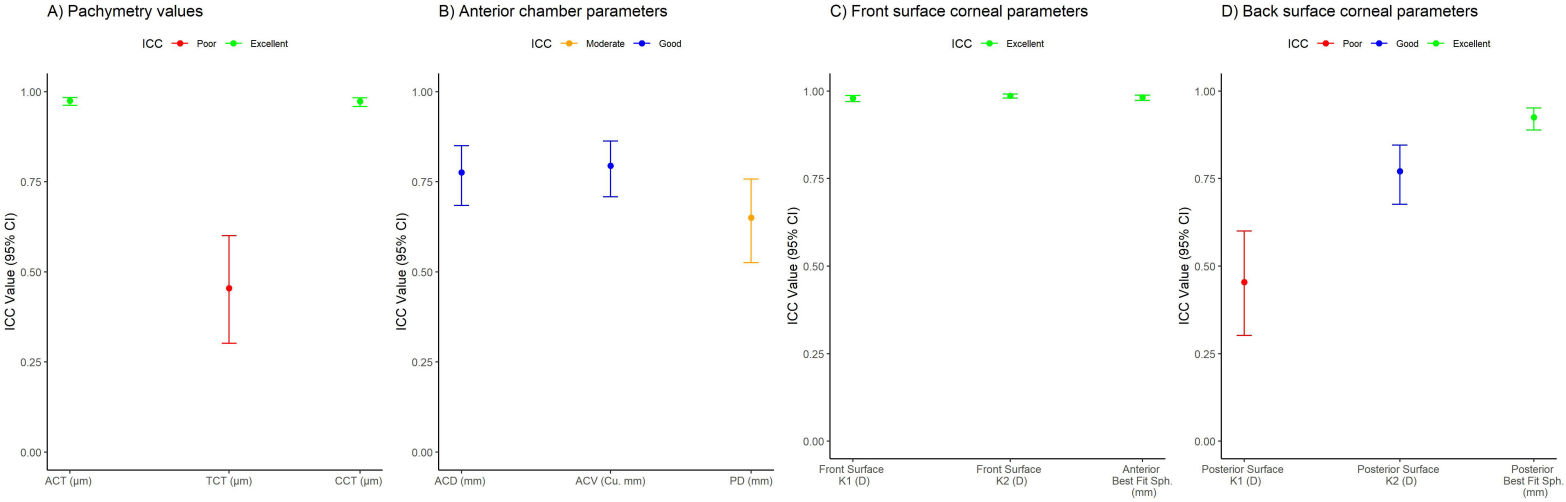
Showing repeatability analysis for all parameters. The dot represents the Intraclass correlation coefficient (ICC) values and error bars represents the 95% confidence interval for ICC.

In a subset of 60 patients, VX650 readings were repeated three times by a single examiner. Between the imaging sessions, the patients were given a break, and then they repositioned their head and chin for the subsequent measurement. Of the 60 patients, 30 had a healthy cornea, 16 had undergone post-refractive surgery, and 14 had keratoconus. The intra class correlation coefficient (ICC) values were calculated for all parameters to evaluate repeatability. Thin corneal thickness and posterior surface K1 showed poor repeatability (**Figure 5**). Pupillary diameter demonstrated moderate repeatability, while the remaining parameters showed good to excellent repeatability (ICC above 0.75).

## Discussion

With the availability of advanced equipment for anterior segment evaluation, comparative studies between devices are just as crucial as understanding each device individually. This is particularly important since patients or surgical candidates may undergo anterior segment assessments using different devices, depending on the equipment available at various ophthalmic centers. In the context of comparing the VX650 and Pentacam, such evaluations are crucial to determine whether the differences in measurements are within acceptable limits and if one device’s results can be reliably substituted or considered interchangeable with the other.

This study compared the corneal and anterior chamber parameters in individuals with keratoconus, post-refractive surgeries and normal corneas using Pentacam and VX650. Most of the parameters measured using VX650 were comparable with Pentacam including measurements from a large group of young adult participants. Corneal thickness is important for various corneal pathologies, IOP measurement and to assess a patient’s eligibility for refractive surgery. In healthy eyes, where the central cornea is typically the thinnest zone, the TCT can closely approximate CCT. However, in corneal ectatic conditions or post-refractive surgery, the TCT may significantly vary from the CCT. In eyes classified as normal, both devices showed close agreement in measurements, with the VX650 reporting slightly higher values for pachymetry, which measures corneal thickness. This difference was statistically significant in various pachymetry parameters such as ACT (-6.79 µm), and CCT (-5.68 µm) in normal corneas, suggesting that clinicians should be aware of this bias when interpreting results, especially in monitoring or diagnosing conditions where corneal thickness is vital. Similar study comparing Pentacam with Sirius in a South Asian population (normal corneas) reported statistically significant differences of −4.45µm and −6.71µm for TCT and CCT respectively (Kumar et al).

In our study, among the eyes that underwent refractive surgery (LASIK, or ICL), the data indicated good agreement between the two devices, though again, with VX650 showing a tendency towards slightly thicker measurements. This finding is particularly relevant for post-surgical monitoring, where accurate corneal thickness measurements are critical in evaluating post-operative recovery and the potential for complications (3, 12). Keratoconus eyes presented a unique challenge, as this condition is characterized by thinning and coning of the cornea. The results highlighted the importance of accurate and reliable measurements in diagnosing and monitoring progression (13) Both devices demonstrated good agreement in keratoconus eyes, although the differences in readings between them highlight the necessity for standardization or device-specific normative data when using these measurements clinically.

While Scheimpflug-based imaging devices like Pentacam, Galilei, and Sirius operate on the same principle, studies have shown that they cannot be used interchangeably, as the anterior segment parameters measured by each device differ, even in healthy corneas. A similar comparison conducted in a South Asian population (the same ethnic group as the current study) demonstrated good repeatability of anterior segment parameters in healthy eyes using Pentacam, Sirius, Orbscan IIz, Corvis, and ultrasound pachymetry (Tomey), and in keratoconus eyes with Pentacam, Galilei, and Sirius (14). However, significant differences in measurements between these devices indicate they are not interchangeable. The VX650 exhibited excellent repeatability in measuring central corneal thickness (CCT). However, thinnest corneal thickness (TCT) displayed poorer repeatability compared to other variables, likely due to patient-related factors such as small movements or blinking, dryness, as well as potential corneal abnormalities.

Corneal curvature measurements are essential for various applications, including contact lens fitting, orthokeratology, and surgical procedures like cataract and refractive surgeries. While the VX650 and Pentacam showed statistically significant differences in front and back surface K1 and K2 values and BFS, the maximum variations were 0.78D, 1.13D and 0.12mm, respectively. Similar discrepancies have been observed in other Scheimpflug devices, underscoring the importance of clinicians carefully considering these variations when planning interventions. The front surface variables demonstrated excellent repeatability. However, the back surface K1 showed poor repeatability, while both back surface K2 and BFS exhibited good repeatability. We anticipate this could be due to alignment issues, or device sensitivity. Our findings suggest that most keratometric values cannot be used interchangeably between the two devices due to variability between the methods. Factors contributing to the variability in corneal topography measurement include examiner training and adherence to the test protocol, disease severity, time between examinations, and patient cooperation. Although the mean differences were less than 0.50D, accurate and consistent corneal measurements are critical for diagnosing and managing conditions like keratoconus and monitoring post-refractive surgical outcomes. Other studies have also reported differences in corneal topography measurements (14, 15). Understanding whether the differences between devices such as Pentacam and VX650, are acceptable is essential to determine if one device’s measurements can be replaced by or used interchangeably with the other. This ensures clinicians choose the most reliable tool for each clinical scenario, ultimately improving patient care and treatment planning.

Accurate anterior chamber depth (ACD) measurements are essential for effective cataract surgery planning, refractive procedures, and the assessment of conditions such as glaucoma. Multiple studies have highlighted variations (range 0.001 to 0.26mm) in ACD measurements among Scheimpflug-based imaging devices, including Pentacam, Galilei, and Sirius (14, 16). In our study, we also observed minor differences in ACD (0.02 mm) between the VX650 and Pentacam, particularly in normal group, while significant differences in anterior chamber volume (ACV) were noted between normal (11.86 cu. mm) and abnormal group (refractive surgery: 24.17 cu.mm and keratoconus: 9.92 cu.mm). ACD and ACV are crucial for assessing suitability and safety in various eye procedures like ICL implantation, cataract surgery, and glaucoma management (angle closures). We had recruited only 3 patients that underwent ICL with an average difference of 0.7 mm (ACD) and 78 cu.mm (ACV). Additionally, pupil diameter (PD) exhibited variability only within the keratoconus group (0.38 mm), underscoring the importance of device selection and interpretation in different clinical contexts. VX650 showed moderate to good repeatability of AC measurements, indicating its reliability for clinical assessments. However, we also emphasize the need for careful interpretation of results, particularly in cases where subtle variations may impact clinical outcomes.

The measurement differences between the VX650 and Pentacam could be attributed to several factors. First, differences in measurement techniques exist, as the Remidio-Visionix VX650 employs a combined approach of Placido-based topography and Scheimpflug imaging to measure corneal thickness, while the Pentacam relies solely on Scheimpflug imaging. Second, measurement zone differences may contribute to variations, as the devices may assess corneal thickness at slightly different locations. Even small differences in the sampled areas (e.g., central vs. slightly paracentral) can lead to consistent measurement discrepancies. Third, device-specific algorithms play a role, as both VX650 and Pentacam utilize proprietary algorithms to calculate various parameters. Differences in how these algorithms process optical properties, such as refractive indices and light reflections, may contribute to the observed variations. Similar discrepancies between devices using different technologies have been reported in other validation studies, as outlined in **Table 1** (14–19).

**Table 1:** Summary of various studies that have compared corneal thickness, front and back surface curvature, and anterior chamber parameters measured by different Scheimpflug-based devices across different ethnic groups.

Study		Current study		Kumar et al., 2019 (14)		Finis et al., 2015 (15)			Anayol et al., 2014 (16)		Abdi et al., 2023 (17)	Baradaran-Rafii et al., 2016 (18)	Qing Wei Zhang et al., 2019 (19)
Devices Compared		Pentacam		Pentacam	Pentacam	Pentacam			Pentacam	Pentacam	Pentacam	Pentacam	Pentacam
		Vs		Vs	Vs	Vs			Vs	Vs	Vs	Vs	Vs
		VX-650		Sirius	OrbScan IIz	Sirius			Sirus	Galilei	Sirius	Galilei	VX-650
Cornea Normal/Abnormal		Normal cornea	Abnormal cornea	Normal cornea		Normal cornea	Regular astigmatism	Keratoconus cornea	Normal cornea		Normal cornea	Normal cornea	Normal cornea
		n = 141 eyes	n = 61 eyes	n = 46 eyes		n = 60 eyes	n = 30 eyes	n = 82 eyes	n = 32 eyes		n= 60 eyes	n= 176 eyes	n=140 eyes
Pachymetry	CCT	-5.68	-0.115	-6.71	-0.41				0.73	13.93		-16	
		P<0.001	P = 0.949	P<0.001	P=1.00				P=1.0	P<0.001		P<0.001	
	TCT	-6.79	-4.11	-4.45	8.34	-33.4	8.96	25.45	-0.03	5.5	3.47		
		P<0.001	P=0.029	P=0.001	P=0.018	P<0.001	P<0.001	P<0.001	P=1.0	P<0.001	P=0.458		
	ACT	-6.79	-4.11			10.09	-30.21	-0.83			-131		
		P<0.001	P = 0.029			P<0.001	P<0.001	P=0.975			P=0.031		
Corneal indices	FS K1	0.37	0.43	0.13	-0.9	0.047	0.054	0.203					-0.2
		P<0.001	P<0.001	P=0.021	P<0.001	P<0.001	P=0.002	P<0.001					P<0.001
	FS K2	0.39	0.48	0.15	0.46	0.043	-0.025	0.084					-0.23
		P<0.001	P<0.001	P=0.005	P<0.001	P<0.001	P=0.625	P=0.059					P<0.001
	FS BFS	-0.02	0.042									0.04	0.04
		P=0.053	P=0.027									P<0.001	P<0.001
	BS K1	0.37	0.43			0.031	0.03	0.49					0.02
		P<0.001	P<0.001			P=0.337	P=0.587	P<0.001					P<0.001
	BS K2	0.39	0.48			-0.098	-0.119	-0.054					0.16
		P<0.001	P<0.001			P<0.001	P=0.001	P=0.662					P<0.001
	BS BFS	0.05	0.01									-0.05	-0.11
		P=0.004	P=0.507									P<0.001	P<0.001
Anterior chamber	ACD	-0.024	0.085	0.05	0.09				P=0.04	P=0.11	0.001	-0.01	0.26
		P=0.048	P=0.04	P=0.001	P<0.001				P<0.001	P<0.001	P=0.851	P<0.001	P<0.001
	ACV	11.86	14.59									78	
		P<0.001	P=0.001									P=0.004	
	PD	-0.01	0.308								1.49	0.57	
		P=0.072	P<0.001								P<0.001	P=0.018	

The Remidio Visionix VX650 stands out as a valuable multimodal device, integrating various tests such as corneal tomography, wavefront analysis, and pachymetry within a single platform. This combination enhances diagnostic capabilities, allowing for comprehensive anterior segment evaluations. Its ability to provide quick and reliable measurements across multiple parameters makes it particularly advantageous in busy clinical settings. Furthermore, the VX650’s moderate to good repeatability in anterior chamber measurements reinforces its reliability for clinical assessments, facilitating informed decision-making for surgical interventions and refractive procedures. Overall, the VX650’s versatility enhances patient care by providing clinicians with a more holistic view of corneal and anterior chamber health. This study’s strengths include a robust sample size of young adult participants, allowing for a comprehensive comparison of anterior chamber and corneal parameters across different eye conditions, such as keratoconus and post-refractive surgeries.

This study adds valuable insights to clinical practice by evaluating the accuracy and reliability of the Remidio Visionix VX650 in real-world clinical settings. It provides evidence on the device’s performance for measuring corneal parameters, offering clinicians a reliable and efficient tool for (specific condition or procedure, e.g., corneal assessment, refractive surgery planning, or diagnostic purposes). The findings support the integration of the Remidio Visionix VX650 into routine practice, which can enhance diagnostic accuracy, improve patient management, and streamline workflows, ultimately leading to more timely interventions and optimized care delivery. However, the study also emphasizes the importance of clinicians not using different devices interchangeably without considering potential calibration or measurement differences, as this may lead to inconsistent results. Standardizing device use within a clinical setting is crucial to ensuring the accuracy and reliability of patient assessments.

Despite its strengths, the study has limitations, including the potential influence of factors such as examiner training, adherence to testing protocols, and patient cooperation, which may affect measurement consistency. The study included only kerataconus and post-refractive surgery cases under abnormal corneas limiting the understanding of the performance of VX650 in other corneal conditions and those with difference anterior chamber measurements such as in glaucoma. Good distribution based on different conditions indicated for anterior segment imaging would provide more understanding of the device. Additionally, while the study focused on a specific ethnic group, the findings may not be generalizable to all populations. The reliance on specific imaging devices also raises concerns about the reproducibility of results across different settings and equipment.

## Conclusion

The Remidio Visionix VX650’s multimodal capabilities further enhance its utility, providing clinicians with comprehensive insights into corneal and anterior chamber conditions, ultimately improving patient management and outcomes. Its adaptability for use in both specialty clinics and comprehensive screening makes it an asset in modern ophthalmic practice. While both VX650 and Pentacam yield valuable data, significant differences in measurements highlight the need for careful interpretation and consideration of device-specific characteristics in clinics.

## Data Availability

The raw data supporting the conclusions of this article will be made available by the authors, without undue reservation.
